# The Welfare of Performing Animals. A Historical Perspective. By David A. H. Wilson. Springer: Berlin, Germany, 2015; 278 pp; $189.00; ISBN 978-3-662-50931-9

**DOI:** 10.3390/ani6110076

**Published:** 2016-11-21

**Authors:** Marthe Kiley-Worthington

**Affiliations:** Centre d’eco-etho Recherche et Education, La Combe Benzaudun sur Bine, Drome 26460, France; marthekileyworthington@gmail.com; Tel.: +33-475532027

Phillips is the editor of this series on scholarly works on animal welfare which are “designed to contribute towards a culture of respect for animals and their welfare by producing learned treatises…”. However, his opinions on the issue of performing animals are evident. He mentions the anthropocentric world in which people live today, and the value that animals have to humans, but then assumes that the only reason for having performing animals in circuses is “humans gaining pleasure from watching animals performing ridiculous tasks” and performing “unnatural acts”. Questions such as whether this is the only motivation for animal acts in circuses and whether *the animals* feel the act is ridiculous are not mentioned. Another question with its origin in the 19th century, is what is an “unnatural act” and does it do harm to the animals to perform “unnatural acts”? Foxes and bears have adapted to live in cities and burrow through dustbins. This is certainly unnatural, but is it harmful to them? Then again, wild dogs in the same cities fend for themselves and rightly regard humans as their major predator since they constantly catch them (and kill them) while their cousins live as companion animals in association with humans and even like them. So which is “unnatural”? Both have learnt to adapt to their situation, surely this is a natural characteristic in any mammal whether considered “wild” or “domestic”. 

There is indeed a need for those interested in furthering the debates to benefit animals in the long run, but I am not sure this book is geared to this aim, although it is an interesting account of one part of human social history. 

Some performing animals in circuses, zoos (of which there are now more and more doing performances), pets, farm animals, horses and all other animals have bad training and bad lives at the hands of humans. But does this mean that no one should have to do with them, see them, admire them, live with them experience them or teach them, that is perform with them? Why, if animals are to be given “respect”, is it applauded and acceptable to use animals for human therapy to be taught to do “unnatural acts”? Animals that help humans (including primates and many others) such as the deaf, the blind, the aged, the immobile or the mentally or physically handicapped, the business person etc., *all perform unnatural acts*. In addition, they may be highly restricted all their lives, yet, their trainers and suppliers are praised, why is that when circuses are hounded on the grounds of “unnatural acts”? 

It is doubtful whether either the animals or I can make a clear distinction between performing animals kept in circuses and performing animals kept for the therapy of humans. Perhaps the title should be “the welfare of performing animals for entertainment only”. But, even here the definition is blurred as Wilson in his introduction states that “training for obedience trials, military purposes, dressage and the like is not dealt with here…” even though most of these animals are taught and perform for entertainment.

In chapter two, the author, David Wilson, traces the training of animals from the Romans, but states that the welfare of performing animals was not seriously considered until the enlightenment. The writings of Aquinas (1225–1274) [1], and Montaigne (1533–1583) [2] are not considered, although, they had a very considerable effect on the culture and understanding of humans′ moral obligations to animals. Wilson states (p. 8) “the medieval world, …was unaware or negligent of the interests of its animals” not so, nor does he mention the classic for training horses written by Xenophon (431–354 BC) [3], who might well turn in his grave at some of the practises used in the approved training of horses used at the Olympics today!

That animals had similar moral rights to humans (that is they were considered moral agents) and consequently were tried by a court of law (Evans [4], first published 1831) is not quoted. This work shows that in the 15th century the mental similarities between humans and other animals were recognized… well before Darwin. There were some interesting cases brought to court for example, even the meat of a cow sentenced to death for causing the death of a woman, was not permitted to be eaten because “ to eat a creature which had become *a peer of man* in blood-guiltless and in judicial punishment, would savour of anthropophagy”. In 1480 a French jurist, Chassenee, made his career by successfully defending in court a bunch of rats who had” feloniously and wantonly destroyed the barley crop”. In the defence, he argues that the non-appearance of his clients in court, was “on the grounds of the length and difficulty of the journey and the serious perils which attended it, owing to the unwearied vigilance of their mortal enemies, the cats…” Although these examples are not strictly of animals performing for human entertainment, this early recognition of their moral status is often unknown in discussions on whether animals are worth respect because they are moral agents.

Some of the relevant history of ideas on animal welfare has been omitted, but, although I do not agree with Wilson’s somewhat biased approach, I learnt a lot about what animals had been trained to do though history, and some of them are astounding. To be fair, Wilson makes sporadic efforts to be just in his judgements on cruelty in training, but because he does not appear to have much practical knowledge of different species and their teaching, this comes across as slightly curious, why is he disapproving of this and not of that? 

Perhaps the most important criticism I have is that he mentions Clever Hans, but omits the most important point. Clever Hans taught himself [5], initially the trainer did not understand what he was doing to correctly answer the question, although he took the credit of course. Like horses trained today, Clever Hans used the “Clever Hans effect” (subliminal visual signals). … Whatever else this is, it is *clever*, something that humans find very difficult, trapped as they are, in their verbal world. 

The author found a report on monkeys who had been taught to perform on the high wire, but instead of considering their remarkable abilities, which the audience certainly did, he emphasises that they were dressed up as humans (in bad taste, but cruel to the monkeys?) and not that they could turn summersaults on the high wire while holding a basket of eggs and without breaking one. Today, cognitive ethologists working with different animals are getting closer in their experiments to teaching many species things that have been known and taught in the circus for some centuries. Usually these scientists are asking the question “can he learn to do it?” Actually, the circus has demonstrated often enough that he can and does, there is knowledge around on learning and indeed teaching in the circuses, not all cruel or in bad taste. Experimental scientists have recently shown that a dog can recognize 300 symbols of objects, but in the 18th and 19th century pigs and dogs learnt almost as many, it seems! (p. 15).

The important point here is that we can learn a lot from teaching animals to perform, and it does not have to be done with violence or punishment as any good animal teacher has always known… through history [6]. This *statement does not condone bad teaching*. We now know, as some others have known from the ancient Greeks, that when training large animals who can easily kill you, cruelty and instilling fear is likely to be the last thing you do; there are many deaths and injuries to prove this point. It is not that that species is “untrainable”, it is the training that is wrong. 

Because training was not often done in public, (because trainers were vain and horded their own acts) it is assumed that training must be cruel. Most of the protests of cruelty in training, as to day, come from people who do not have daily contact with those animals and is surmised rather than witnessed. Wilson quotes writers who believe cruelty is necessary in training; seeing a tiger caress its keeper (p. 24) the reporter writes “the cruel means by which the fiercest of beasts is taught all the servility of a fawning spaniel” or lower: “when I reflect on the cruelty that must necessarily be used to procure these artificial monsters”, this is a pig who is proficient as a dog or a horse in exhibition, a monster! Any wildlife warden or pig keeper who is worth his salt, is aware their animals can learn to do these things and more, not by beating them or hurting them. Perhaps we need people with serious years of practical knowledge to teach todays public to stop them being gripped in the belief in animals having inferior mental inabilities and therefore unable to learn or perform without punishment. This is a left over belief of the dualism of Decartes (16th century.) Ironically, perhaps it is this theory in the mental differences between animals and humans that lies behind the beliefs of most of the anti-animal teaching and animal apartheid activists. 

My take home message from this book therefore is how amazingly physically and mentally able many of these species are, they have shown that they can learn an enormous and unlikely range of behaviours for performances in circuses. Although some of the training was cruel, it does not have to be.

There are interesting chapters concerning the parliamentary committees and debates on performing animals, and the usual arguments about “dignity” when animals perform “unnatural acts”. Whether the animals have the same idea of “dignity” as the Cultural Elite and Animal Welfare Activists is unlikely. If they do, then they certainly have the same mental attributes, one of them being to learn! 

The final chapter entitled “animal performance and applied science” is a fair assessment of the various views that have been published by scientists over the last half century. The debates will continue, even if there are no more so called “wild animals” in circuses in some countries. What we need is good science on animal teaching, and the development of Animal Educational Psychology (e.g., [4]) before we are convinced that animals or humans should never be used for performance or entertainment. 

I do wonder what happened to all the tigers and lions that have been successfully bred in circuses for generations. For their sakes, I hope that they have not been thrown out into the non-existent “wild” where we know captive born animals have little hope of survival and will suffer trauma as a result of their life time experiences. Perhaps worse for them is to be dumped in a zoo, forced to associate closely with others they do not know and they have nothing else to do. Lifetime experiences are important, whether you are a circus elephant, a police horse, a tribal Kenyan, or a so called “disinterested” scientist… Our lifetime experiences matter and control much of what we can learn, our beliefs and ideas, how and if we can adapt to different situations and the choices and decisions we make whatever mammal we are, even if a “scientist” or historian… like it or not.

This book is a clearly written English speaker’s historical perspective on mostly the evils that may have been used to train circus animals and the curiously muddled thinking that has dogged the parliamentary debates to eventually ban “wild” animals in circuses. But, despite the considerable unnecessary suffering of many of these animals, what they learnt and did with humans was often extraordinary. We might have been able to combine the wild animals’ needs and ours in order to learn more about them, if circus training and the living accommodation for the animals had improved and been allowed to continue, but no longer since they are banned. The skills will be lost within a generation, although some may have to be reconstructed in the future to help with the keeping of large mammals almost all of whom live in large managed zoos: “nature reserves”. May be non-English speaking countries will continue with their animals in circuses so perhaps our duties are to *improve* their animal teaching and keeping to ensure that the animals do not suffer and some of the best of the circus trainers knowledge remains with us. 
